# Supplementary material for: Tergitol Based Decellularization Protocol Improves the Prerequisites for Pulmonary Xenografts: Characterization and Biocompatibility Assessment
Source: Polymers (Basel). 2023 Feb 6;15(4):819. doi: 10.3390/polym15040819 (PMC9967102; doi:10.3390/polym15040819)
Supplement: Supplementary file 1 [file polymers-15-00819-s001.zip › polymers-2170728-supplementary.pdf]

ANTIBODY	CAT. NUMBER	COMPANY	DILUTION
Anti-Collagen I	C2456	Sigma	Mouse, 1:100
Anti-Collagen IV	AMab6586	Abcam	Rabbit, 1:200
Anti-Elastin	AB2161D	Abcam	Rabbit, 1:50
Anti-Laminin	L9393	Sigma	Rabbit, 1:100
Anti-Alpha-gal	LS-C63415	LS Bio	1:10
DAPI	R37606	ThermoFischer Scientific	10μg/ml
Alexa Fluor 488 (anti-Rabbit)	A11008	Invitrogen by ThermoFisher	1:200
Alexa Fluor 555 (anti-Mouse)	A21422	Invitrogen by ThermoFischer	1:300

Table S1. List of primary and secondary antibodies used in the study in correspondence with their dilutions used.

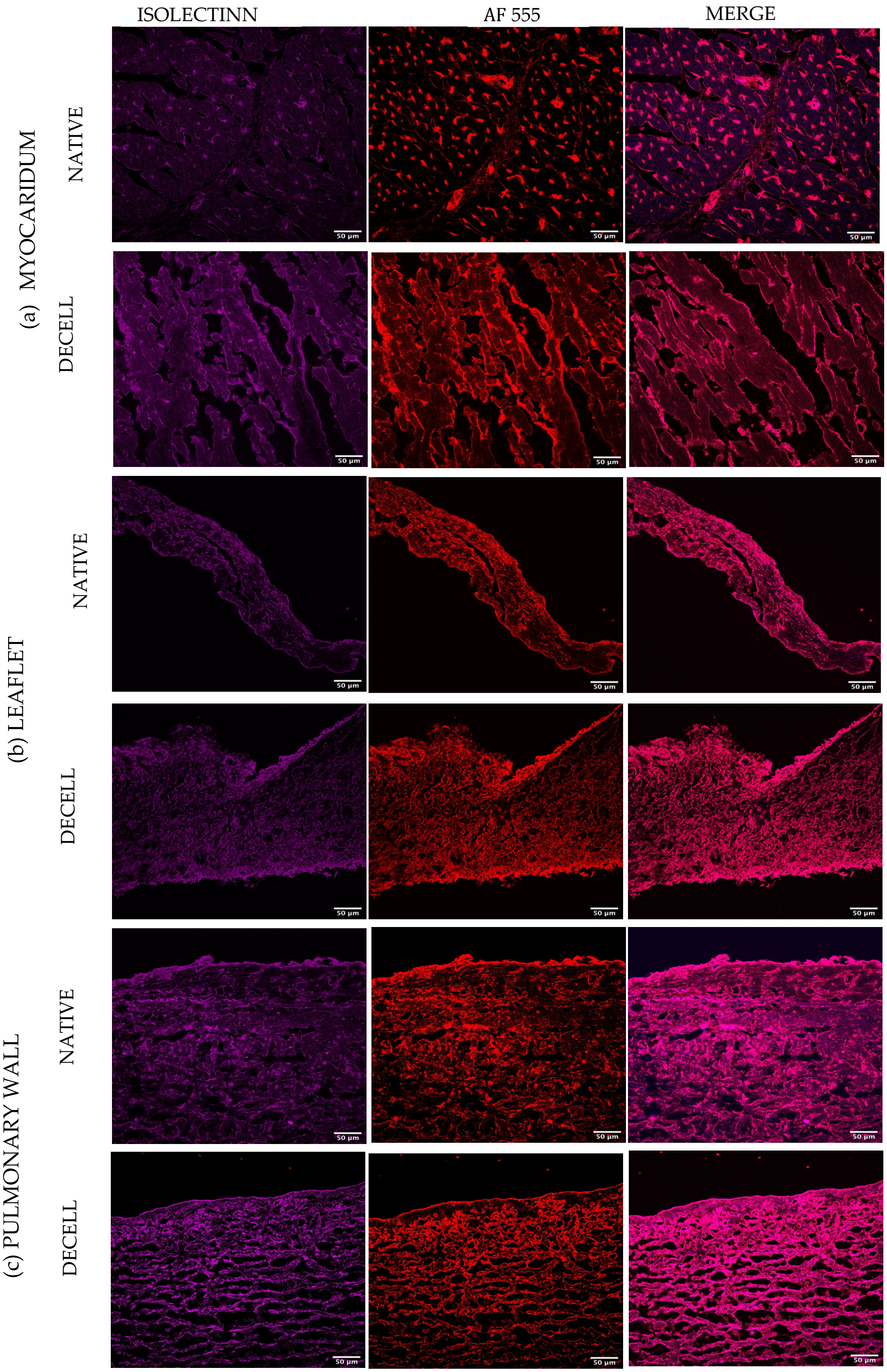

Figure S1. Alpha gal detection with isolectin and anti- alpha gal antibody in native and decellularized a) myocardium, b) leaflet and c) pulmonary wall. Confocal imaging was performed using microscope Zeiss Axio Observer LSM 800 at 20x magnification. Images were processed with Fiji software. Alpha gal signal was reduced but not removed in decellularized pulmonary tissues.

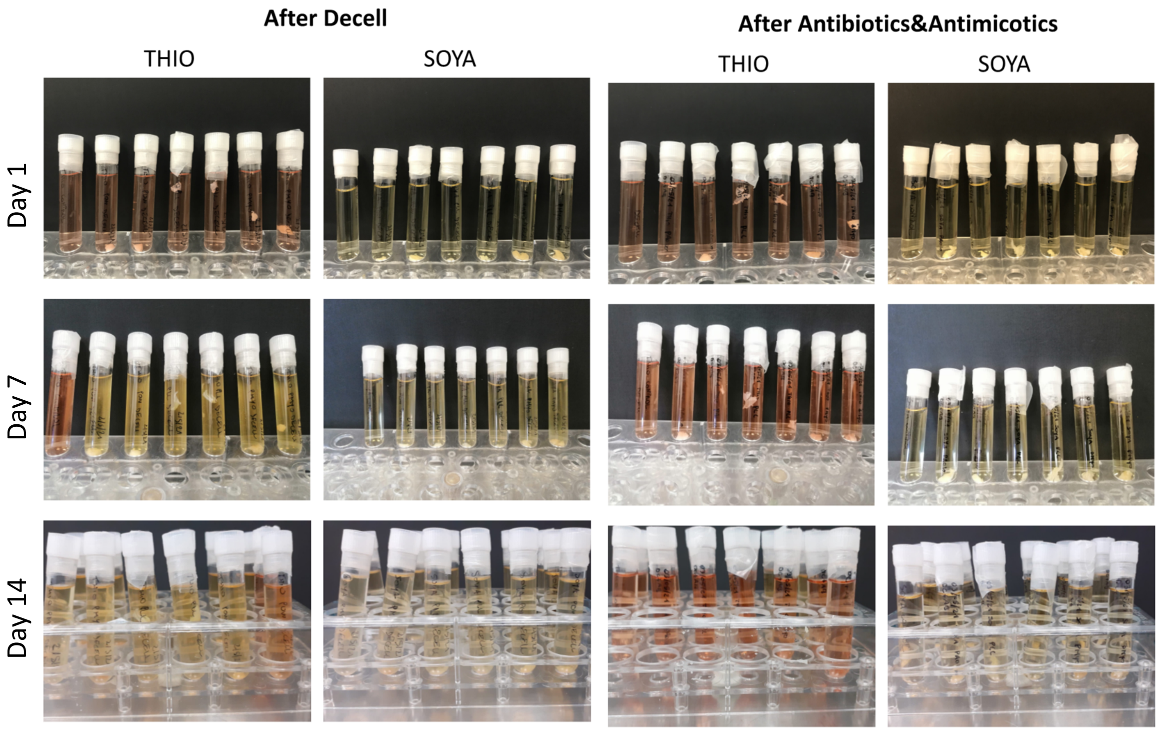

Figure S2. Sterility assessment of native and decellularized plus decontaminated (with Antibiotic and Antimycotic solution) samples from day 0 to day 14.
